# High Sensitivity of Mobile Phone Microscopy Screening for *Schistosoma haematobium* in Azaguié, Côte d’Ivoire

**DOI:** 10.4269/ajtmh.22-0527

**Published:** 2022-12-12

**Authors:** Jean T. Coulibaly, Kigbafori D. Silue, Maxim Armstrong, María Díaz de León Derby, Michael V. D’Ambrosio, Daniel A. Fletcher, Jennifer Keiser, Karla Fisher, Jason R. Andrews, Isaac I. Bogoch

**Affiliations:** ^1^ Unité de Formation et de Recherche Biosciences, Université Félix Houphouët-Boigny, Abidjan, Côte d’Ivoire;; ^2^ Centre Suisse de Recherches Scientifiques en Côte d’Ivoire, Abidjan, Côte d’Ivoire;; ^3^ Department of Bioengineering, University of California, Berkeley, Berkeley, California;; ^4^ Biological Systems and Engineering Division, Lawrence Berkeley National Laboratory, University of California, Berkeley, Berkeley, California;; ^5^ Chan Zuckerberg Biohub, San Francisco, California;; ^6^ Swiss Tropical and Public Health Institute, Allschwil, Switzerland;; ^7^ University of Basel, Basel, Switzerland;; ^8^ Divisions of General Internal Medicine and Infectious Diseases, Toronto General Hospital, University Health Network, Toronto, Canada;; ^9^ Division of Infectious Diseases and Geographic Medicine, Stanford University School of Medicine, Stanford, California;; ^10^ Department of Medicine, University of Toronto, Toronto, Canada

## Abstract

Schistosomiasis infections continue to impact African settings disproportionately, and there is an urgent need for novel tools to evaluate infection control and elimination strategies at the community level. Mobile phone microscopes are portable and semiautomated devices with multiple applications for screening neglected tropical diseases. In a community-based schistosomiasis screening program in Azaguié, Côte d’Ivoire, mobile phone microscopy demonstrated a sensitivity of 85.7% (95% CI: 69.7–95.2%) and specificity of 93.3% (95% CI: 87.7–96.9%) for *Schistosoma haematobium* identification compared with conventional light microscopy, and 95% sensitivity (95% CI: 74.1–99.8%) with egg concentrations of five or more per 10 mL of urine. Mobile phone microscopy is a promising tool for schistosomiasis control and elimination efforts.

## INTRODUCTION

Schistosomiasis continues to be a driver of morbidity and mortality in low-income countries, with the greatest burden occurring in sub-Saharan Africa.^1^ The infection disproportionally affects impoverished communities and children, primarily in rural locations.^2^ The WHO’s schistosomiasis control initiatives call for mass drug administration (MDA) campaigns to treat all school-age children without prior diagnosis if the prevalence of infection is greater than 10% in a community. The WHO also highlights an urgent need for tools to help monitor and evaluate such MDA programs.^3^

Mobile phone microscopes are a promising tool for the diagnosis and screening of neglected tropical infections.^4^ Mobile phone microscopy may help identify regions eligible for MDA, and they have attributes that may be useful in monitoring and evaluating schistosomiasis control programs given that they are portable, battery powered, relatively easy to use, and provide a result in real time (often at the point of data collection).^5^ Here we evaluate a mobile phone microscope, known as the SchistoScope,^6^ coupled with a novel urine filtration system for schistosomiasis screening in the Azaguié region of Côte d’Ivoire.

## METHODS

Ethical permission for this study was granted by the Center Suisse de Recherches Scientifiques en Côte d’Ivoire, Abidjan, Côte d’Ivoire (#054-19) and the University Health Network, Toronto, Canada (REB #14-8128). Permission was also granted by the local Health District officer. School-age children between 5 and 14 years were invited to participate, and both signed parental consent and the children’s assent were required for inclusion. This study was conducted in March 2020 and performed in the context of a larger screening and treatment program evaluating the prevalence of schistosomiasis in school-age children in the region. All children at identified schools were offered inclusion in the study, and children had to provide a single urine sample to be included.

Urine samples were collected between 10:00 am and 2:00 pm, then processed and examined that same day. Urine samples were first shaken, and 10 mL was then removed in a syringe and pressed through a 20-micron filter paper (Sefar AG, Heiden, Switzerland). The filter paper was then removed and positioned on a glass slide, with a single drop of Lugol’s iodine placed on the filter paper. These samples were evaluated for *Schistosoma haematobium* eggs by a laboratory technician under 5× and 20× magnification.^7^ The presence or absence of *S. haematobium* eggs was noted and quantified if positive. Ten percent of all samples were reevaluated by an expert microscopist for quality control.

**Figure 1.**
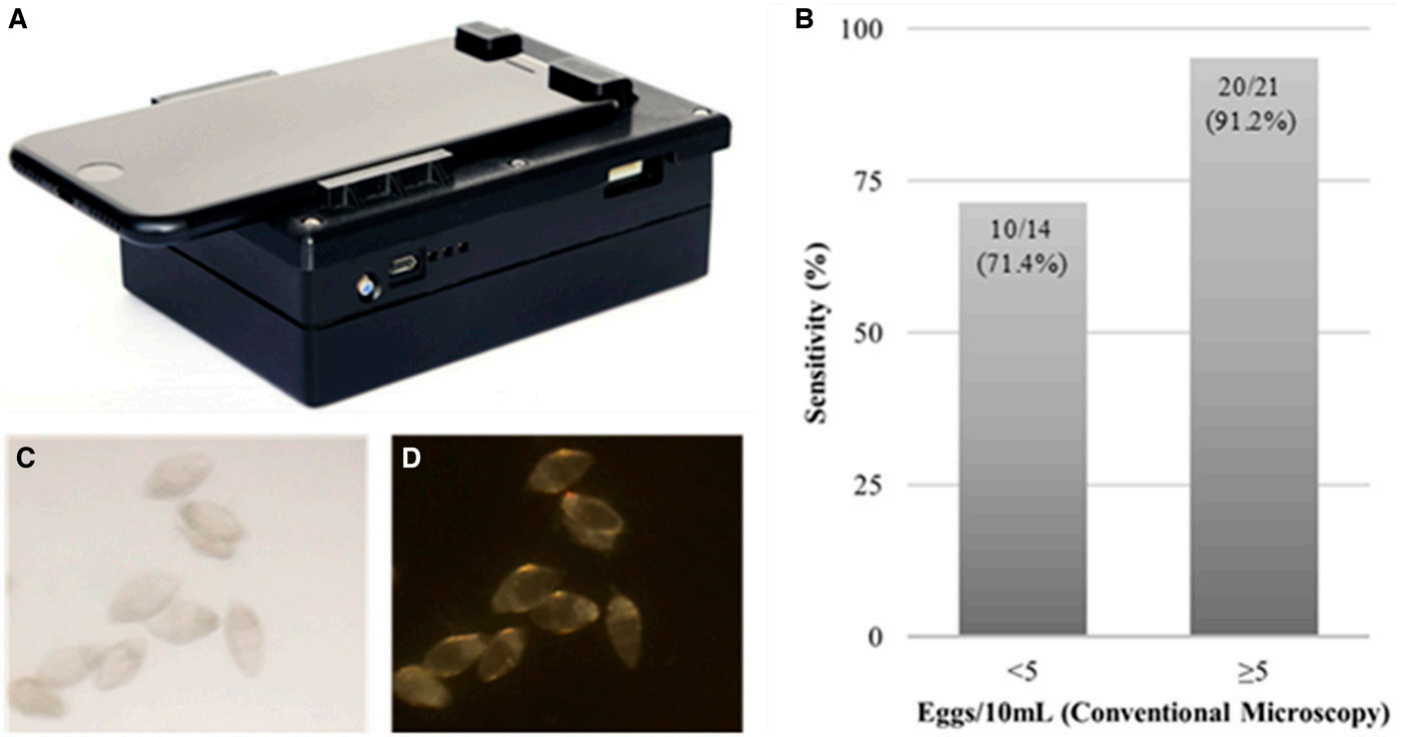
(**A**) The SchistoScope mobile phone microscope, consisting of a mobile phone mounted on a three-dimensional printed device that magnifies and illuminates samples (photo credit: Daniel Ehrenworth). (**B**) Sensitivity of mobile phone microscopy for *S. haematobium* identification with urine egg concentrations greater or less than five eggs per mL (**C**). *S. haematobium* eggs visualized by mobile phone microscopy using light field illumination. (**D**) *S. haematobium* eggs visualized by mobile phone microscopy using darkfield illumination.

In tandem, 10 mL of urine from the same container was pressed through a cartridge designed to trap *S. haematobium* eggs, which was then placed in the mobile phone microscope (Figure 1A). The cartridge has a rectangular cross-section that tapers down from a height of 200 to 20 µm, trapping the eggs in a region that can be directly imaged on a microscope.^6^ The SchistoScope used for imaging is a modification of the LoaScope^8^ with added illumination methods and an updated mobile phone. Each cartridge was imaged using the camera function of the mobile phone, an iPhone 8 (Apple, Cupertino, CA), under light field, dark field, and florescence lighting. Images were collected, evaluated, and stored on the mobile phone device. Any smartphone device with a camera function may be used; however, the SchistoScope was designed around the iPhone 8, and slight modifications would be needed to accommodate other devices. *S. haematobium* eggs were noted to be present or absent and, if present, were quantified and entered into a database. Microscopists using the mobile phone and conventional microscopes were blinded to any prior results of the samples they were evaluating. We calculated sensitivity and specificity of the cartridge-based filtration device paired with mobile microscopy, compared with the reference standard of conventional microscopy, reporting exact binomial CIs. We evaluated correlation in egg counts using Pearson’s *r*. All statistical analyses were performed using R (version 4.0.3).

## RESULTS

One hundred and seventy individuals provided urine specimens and were included in this study. Thirty-five (20.6%) were found to contain *S. haematobium* eggs by conventional microscopy; only three samples were found to have ≥ 50 eggs per 10 mL, meeting WHO criteria for a high-burden infection.^9^ Compared with conventional microscopy, urine filtered through cartridges and evaluated via mobile phone microscopy demonstrated a sensitivity 85.7% (95% CI: 69.7–95.2%) and specificity of 93.3% (95% CI: 87.7–96.9%). The mobile phone microscope missed five total infections out of the 35 samples with *S. haematobium* eggs detected by conventional light microscopy, and four of those samples had egg counts of fewer than five eggs per 10 mL (Figure 1B–D). Mobile phone microscopy had 95% sensitivity (95% CI: 74.1–99.8%) with *S. haematobium* egg concentrations of five or more per 10 mL. Pearson’s correlation among samples positive by at least one test was 0.92.

## DISCUSSION

We demonstrate the high sensitivity and specificity of mobile phone microscopy for the diagnosis of *S. haematobium* infection relative to conventional light microscopy, the current field gold standard, in an endemic community.

The SchistoScope was able to identify most cases of *S. haematobium*, infrequently missing very low burden infections, most of which had fewer than five eggs per 10 mL of urine. Although any identifiable helminth eggs are concerning, there is a marked reduction in morbidity in those with lower egg burdens.^10^ The SchistoScope may be an ideal tool for rapid screening of *S. haematobium* in school- or community-based settings. These devices are portable, battery powered, and designed to filter and analyze urine samples quickly, at the point of collection.^4^ Ongoing work to integrate artificial intelligence on to mobile phones for the automation of helminth egg identification and quantification would make these pragmatic screening tools.^6^^,^^11^^,^^12^ Combining mobile phone microscopy (for *S. haematobium*) with the point of care circulating cathodic antigen (POC CCA) test (for *Schistosoma mansoni*) would allow for rapid schistosomiasis screening in a region while requiring only 10 mL of urine, negating the need for time and resource consuming stool collection and processing. A mobile microscopy and POC CCA screening platform is a potential solution to the WHO’s recent call to action for better tools to identify, monitor, and evaluate schistosomiasis MDA control efforts.^3^ One concern requiring ongoing vigilance are the recent reports of inconsistent sensitivity of POC CCA tests, especially in low-prevalence settings.^13^ Still others are finding the tests reliable, and there are emerging urine-based tests that show promise as well (e.g., the up-converting phosphor lateral flow circulating anodic antigen assay).^14^ POC CCA validity will require close follow-up should these tests be integrated into future screening programs.

Incremental modifications to the engineering and design of mobile phone microscopes over the past few years have improved the diagnostic operating characteristics for infections of global health significance.^4^ Further, innovations in sample preparation in field settings has also enabled more flexibility and creativity for microscope design. For example, using injection-molded cartridges for urine filtration in this study allowed for a novel mobile microscope design to detect high-quality digital images while veering from traditional method of visualizing samples on glass slides.^6^ Glass slides are breakable, and it may be challenging to preserve samples, whereas the cartridges trap *S. haematobium* eggs and are comparatively more robust. Coupling novel sample processing techniques with improved image detection allows for greater flexibility in designing sturdy digital microscopes for use in field settings, while adhering to the WHO standard of filtering 10-mL volume of urine for processing. Still, these novel field methods must be validated in larger trials and compared with field gold standards.

Mobile phone microscopy is a sensitive and specific diagnostic for *S. haematobium*, and ongoing work to automate this process and couple it with POC CCA may help with evaluating and monitoring schistosomiasis control efforts.
